# Influenza Vaccination in Children During the First Two Seasons of Routine Vaccination Programs (2023–24 and 2024–25) in Central Catalonia, Spain: A Retrospective Study

**DOI:** 10.3390/ijerph23010032

**Published:** 2025-12-24

**Authors:** Sílvia Burgaya-Subirana, Mònica Balaguer, Laia Sola Reguant, Anna Ruiz-Comellas

**Affiliations:** 1 Pediatrics Department, EAP Manlleu, Institut Català de la Salut, Gerència d’Atenció Primària i a la Comunitat de la Catalunya Central, C/Castellot, 17, 08560 Manlleu, Spain; sburgaya.cc.ics@gencat.cat; 2 Facultiy of Medicine, University of Vic-Central University of Catalonia (UVic-UCC), 08500 Vic, Spain; aruiz.cc.ics@gencat.cat; 3 Health Promotion in Rural Areas Research Group, Fundació Institut Universitari per a la recerca a l’Atenció Primària de Salut Jordi Gol i Gurina, 08242 Manresa, Spain; 4 Pediatric Intensive Care Unit, Hospital Sant Joan de Déu Barcelona, Passeig Sant Joan de Déu, 2, 08950 Esplugues de Llobregat, Spain; 5 Research Department, Institut Català de la Salut, Gerència d’Atenció Primària i a la Comunitat de la Catalunya Central, Carrer de Soler i March, 6, 08242 Manresa, Spain; 6 Medicine Department, EAP Sant Joan de Vilatorrada, Institut Català de la Salut, Gerència d’Atenció Primària i a la Comunitat de la Catalunya Central, Avinguda del Torrent del Canigó, 0, 08250 Sant Joan de Vilatorrada, Spain

**Keywords:** influenza vaccine, children, vaccination coverage

## Abstract

**Highlights:**

**Public health relevance—How does this work relate to a public health issue?**

- The World Health Organization (WHO) estimates that every year 1 billion people contract the flu, of which 3–5 million have serious symptoms and the number of deaths from respiratory diseases related to influenza infection is between 290,000 and 650,000.
- In Spain, from the 2023–2024 season, the influenza vaccine has become part of the routine vaccination program for children from 6 to 59 months.

**Public health significance—Why is this work of significance to public health?**

- This work assesses the coverage and the adherence of influenza vaccination in children after the introduction of the influenza vaccine into the routine vaccination program in Spain for children under 5 years of age.
- It also attempts to identify the risk factors associated with influenza vaccination in all age groups (under 5 years and from 5 to 14 years with chronic pathologies).

**Public health implications—What are the key implications or messages for practitioners, policy makers and/or researchers in public health?**

- Both the coverage and the adherence to influenza vaccination in children are very low, despite the introduction of a routine influenza vaccination program in children from 6 to 59 months.
- Factors associated with influenza vaccination in children aged 6–59 months included immigrant origin, urban residence, the presence of multiple risk factors, and specific underlying chronic conditions.

**Abstract:**

Vaccination is the primary method of preventing influenza. During the 2023–24 season, the influenza vaccination for all children between 6 and 59 months was introduced for the first time in Spain. To assess the coverage and adherence of influenza vaccination in childhood during the first two seasons of a vaccination program, as well as to identify the factors associated with influenza vaccination in all children under 5 years of age and those from 5 to 14 years of age with risk factors. Retrospective observational study. All children eligible for the flu vaccine in Central Catalonia during the 2023–24 and 2024–25 seasons were included. A total of 39,937 children were studied. Of these, 79.1% had not been vaccinated for influenza in either of the seasons studied. Influenza vaccination coverage in childhood was 18.1% and 19.3% in the 2023–24 and 2024–25 seasons, respectively. In the 6- to 59-month age range, coverage was 19.1% and 28.9% in the 2023–24 and 2024–25 seasons, respectively. The adherence to vaccination in both seasons was 17%. Some variables (being a non-native person, living in an urban area, having more than one risk factor, or certain underlying conditions) were associated with the influenza vaccination. Coverage and adherence to influenza vaccination in childhood are very low, despite the implementation of a routine influenza vaccination program.

## 1. Introduction

Influenza is a very common infection in children and has a large impact on public health [1,2]. It is caused by orthomyxovirus influenza A, B, or C. Viruses A and B are the causes of annual epidemics, and the A virus has caused global pandemics. Although most of the time the symptoms are mild (sudden catarrhal onset with high fever, cough, mucus, and myalgia), it can sometimes have complications and serious symptoms [2,3,4]. The World Health Organization (WHO) estimates that every year 1 billion people contract influenza, 3–5 million of which have serious symptoms, and the number of deaths from respiratory disease related to this infection is between 290,00 and 650,000 [3]. It has been shown that the pediatric population is the most affected by this infection and is the main transmitter of the virus [4,5]. In line with these findings, in Spain, the highest incidence rate for influenza in the 2021–22 and 2022–23 seasons was observed in the 0- to 4-year-old group (1500 cases and 1100 cases per 100,000 inhabitants, respectively), followed by the 5- to 14-year-old group (400 cases and 1000 cases per 100,000 inhabitants, respectively) [6,7]. Hospitalizations for influenza in the same period were the highest in elderly people, followed by the 0- to 4-year-old group [6,7]. Therefore, children under 5 years of age are the most susceptible to influenza and the most vulnerable to serious infection [8]. Vaccination is considered one of the best ways to prevent influenza, and it can reduce the duration of hospital admissions and the risk of severe cases and deaths [5,6,7,8,9]. Several studies have shown that influenza vaccination at the pediatric age can not only protect children but also the entire community, and it contributes to reducing the incidence of influenza in the general population [10,11,12]. Despite this evidence, childhood influenza vaccination coverage in Spain is very low [5,13,14,15,16,17,18,19,20].

In Spain (and, therefore, in Catalonia), until the 2022–23 season, pediatric influenza vaccination was recommended for all children over 6 months of age with any high-risk pathology for complications [Table 1] [21,22], but from the 2023–24 season, and following the recommendations of the Spanish Ministry of Health and the WHO, they were extended to all children under 5 years of age and any children over this age with risk factors [23,24,25]. The influenza vaccine has become part of the routine vaccination calendar.

The objectives of this study are to evaluate the coverage and adherence to childhood influenza vaccination in Central Catalonia during the first two seasons (2023–24 and 2024–25) of routine childhood influenza vaccination, and to identify the factors associated with influenza vaccination both in children under 5 years of age and in children aged from 5 to 14 years who had risk factors.

## 2. Materials and Methods

### 2.1. Study Design and Participants

Retrospective observational study conducted in the health region of Central Catalonia. This is a region located in the northeast of Spain that provides health coverage to about 530,000 people in total and to about 43,000 children from 0 to 14 years.

The study included all children between 6 and 59 months and children between 5 and 14 years of age with any risk factor for secondary influenza complications; that is, all children eligible for the influenza vaccination during the 2023–24 and 2024–25 seasons. For each influenza season, vaccination coverage denominators were defined as all children who met the eligibility criteria at that specific season (children aged 6–59 months and children aged 5–14 years with underlying conditions). Eligibility was assessed independently for each season; therefore, children could be included in one or both seasons, depending on their age and comorbidity status at each seasonal time point. Children younger than 12 months could only contribute to one season, and children with underlying conditions were included only in seasons in which the comorbidity was present. Numerators were defined as the number of eligible children who received the influenza vaccine during the corresponding season.

The data were obtained from the electronic database of the Catalan Institute of Health (ICS), the public health organization that provides health services in Catalonia.

### 2.2. Variables

The variables analyzed for each patient were the following: age, sex, place of residence (rural or urban), education of both parents, influenza vaccination, country of origin (Spain or others), number of risk factors, risk factors for receiving influenza vaccination, and adherence to influenza vaccination.

To classify the place of residence in rural or urban areas the number of inhabitants of each population was considered; an area was considered rural or semi-urban if it had less than 10,000 inhabitants and urban if it had more than 10,000.

The variable “parental education” has been extracted from registries.

The risk factors were grouped into the following 11 categories in order to study them closely: asthma, heart disease, chromosomal and metabolopathies, diabetes, hemoglobinopathies and clotting disorders, immunosuppression and neoplasms, neuromuscular and neurological diseases, celiac disease, nephropathies and hepatopathies, obesity, and miscellaneous conditions.

Having been vaccinated in both seasons consecutively was considered complete adherence to influenza vaccination. To calculate this variable, those patients who had not been able to receive the vaccination in two consecutive seasons, due to age, were excluded.

### 2.3. Statistical Analysis

A descriptive analysis of the characteristics of the child population included in the study was conducted. Categorical variables were summarized by absolute frequencies and percentages, while quantitative variables were described by means and standard deviations (SD) in the case of normal distribution, or with median and interquartile range (RIQ) in the case of non-normal distribution, which was determined by the Shapiro–Wilk test.

Vaccination coverage was calculated as the percentage of vaccinated children compared to the total number of eligible children per season. Adherence to vaccination was defined as the proportion of children vaccinated in both seasons compared to those vaccinated in at least one.

For the comparison of proportions between groups (e.g., coverage according to age range, origin, or presence of pathologies), the *X*^2^ test (Chi-square) or Fisher test was used when the expected frequencies were less than 5. To compare quantitative variables between two groups, Student’s *t*-test was used for independent samples or, in the case of non-normal distribution, the Mann–Whitney U test.

To analyze the factors associated with influenza vaccination, a multivariate logistic regression model was applied. The dependent variable was the condition of having been vaccinated (yes/no) and the independent variables included age, sex, immigrant origin, number of risk factors, and presence of basic pathologies. The odds ratio (OR) was calculated with the corresponding confidence intervals at 95% (CI 95%).

The statistical significance was established with a value of *p* < 0.05. The analysis was carried out with R statistical software (version 4.3.1).

Statistical analysis were performed with program R version 4.2.1 (R Foundation for Statistical Computing).

### 2.4. Ethical Considerations

The research was carried out in accordance with the Helsinki Declaration and Spanish national and institutional legislation concerning clinical research and the protection of personal data. The data collected from the database of the Catalan Institute of Health were pseudo-anonymized by the technical area of this body so that no member of the research team could identify the participants. Therefore, no informed consent was required.

The study was approved by the Ethics Committee IDIAP Jordi Gol (code: 23-/139; date of approval: 26 July 2023).

## 3. Results

### 3.1. Characteristics of the Sample

Out of the 39,987 children studied, 53.3% (21,330) were male. Around 40% (16,003) were between 6 months and 2 years and 69.7% (27,875) lived in rural or semi-urban areas. A total of 80.7% (26,696) were of Spanish origin; 79.1% (31,621) had not been vaccinated in either of the two seasons studied. With regard to risk factors, most had no underlying conditions (72.4%). The most prevalent risk factors were obesity (15.6%), followed by asthma (8%). Table 2 shows the clinical and demographic characteristics of children eligible for influenza vaccination during the 2023–24 and 2024–25 seasons.

### 3.2. Differences Between Vaccinated and Unvaccinated Children by Age Groups

As for the age group from 6 to 59 months, male sex predominates (53% vs. 51.1%; *p* 0.005) in the vaccinated group, as was also observed for children from 6 to 24 months (64.6% vs. 50.6%; *p* < 0.001) and those living in urban area (37% vs. 27.8%; *p* < 0.001). In general, educated parents vaccinate more. Children from other countries predominate in the vaccinated group (24.1% vs. 19.7%; *p* < 1 0.001), as do those with risk factors. In terms of underlying conditions, statistically significant differences were observed between vaccinated and unvaccinated children in some of the variables (asthma, heart disease, hemoglobinopathies and clotting disorders, neurological and neuromuscular diseases, and nephropathies and hepatopathies). In regard to the seasons, more vaccinations were observed in the 2024–25 season compared to the 2023–2024 season (57.1% vs. 46.1%; *p* < 0.001). Table 3 shows the descriptive and comparative analysis between vaccinated and unvaccinated children by age groups (<5 years and from 5 to 14 years).

In the subgroup of 5- to 14-year-olds, there are no statistically significant differences between vaccinated and unvaccinated children in the sex, country of origin, and season variables. In the group of vaccinated children, those living in an urban area continue to predominate (35.8% vs. 30.6%; *p* < 0.001). Regarding the education levels of parents, fathers with higher secondary school, third level, or university education and mothers with third level or university education predominate in the vaccinated group. Unlike the previous group where most vaccinated children had only one risk factor, children with two or more risk factors were the majority in this group (13.6% vs. 3.95%; *p* < 0.001). In terms of underlying conditions, statistically significant differences were observed between vaccinated and unvaccinated children in some variables; children with asthma, diabetes, hemoglobinopathies and blood clotting disorders, immunosuppression and neoplasms, neuromuscular and neurological diseases, celiac disease, nephropathies and hepatopathies, and miscellaneous diseases predominated in the vaccinated group. However, regarding the heart disease variable, statistically significant differences between the vaccinated group and the unvaccinated group had disappeared. In the group of non-vaccinated children, the majority were between 11 and 14 years (53.8% vs. 48.2%; *p* < 0.001), lived in rural areas (69.4% vs. 64.2%; *p* < 0.001), and were classified as having obesity (65.6% vs. 21.9%; *p* < 0.001) [Table 4].

### 3.3. Coverage and Adherence to Influenza Vaccination

In Table 5, Table 6 and Table 7 we can see influenza vaccination coverage in childhood by age group. In general, these were low in all seasons and across all age ranges. Note that, although influenza vaccination in childhood (6 months to 14 years) during the 2023–24 and 2024–25 seasons was 18.1% and 19.3%, respectively, in the 6- to 59-months age range in the second season (2024–25), coverage was 7.7% higher than in the previous season, with a statistically significant difference (coverage of 19.09% in the 2023–24 season and 26.82 in the 2024–25 season).

As for adherence, only 17% of children (6 months to 14 years) were vaccinated in the two seasons consecutively (excluding children who could not be vaccinated during the two seasons consecutively because of age).

### 3.4. Variables Associated with Influenza Vaccination

The results of bivariate and multivariate analysis are shown in Table 8 and Table 9. In the bivariate analysis of the 6- to 59-month-old group, male sex was strongly associated with vaccination compared to female sex (OR: 1.08 [1.02–1.14]). In addition, children of Spanish origin were less likely to be vaccinated than non-native people (OR 0.77 [0.72–0.82]), and children living in urban areas were more likely to be vaccinated than rural dwellers (OR 1.53 [1.44–1.62]). Having risk factors is also positively associated with vaccination to a significant degree (the more the risk factors, the more association with influenza vaccination OR 3.94 [1.41–11.2]). Lastly, the risk factors that appear to be the greatest predictors of vaccination were nephropathies and hepatopathies (OR 5.91 [1.79–22.6]), hemoglobinopathies and coagulation disorders (OR 4.40 [1.93–10.3]), diabetes (OR 4.34 [1.02–12.2]), miscellaneous diseases (OR 4.06 [2.24–7.43]), and asthma (OR 3.60 [2.18–5.05]). As for heart disease, it also had a statistically significant positive relation to influenza vaccination (OR 1.59 [1.18–2.11]), although to a lesser extent than the other underlying conditions listed above.

In the multivariate analysis of children aged 6 to 59 months, the results are similar to those of the bivariate analysis, but statistical significance is lost in the sex; ≥2 risk factor variables; and the hemoglobinopathies, clotting disorder, and miscellaneous variables.

Therefore, we can say that in the group of children from 6 to 59 months, the country of origin, rural addresses, the number of risk factors, and some risk factor variables had statistically significant associations with influenza vaccination for this age group.

In the bivariate analysis of the variables associated with vaccination in children aged 5 to 14 years with risk factors, those related to vaccination were rurality (living in an urban area is associated with higher influenza vaccination) (OR 1.26 [1.13–1.41]), number of risk factors (having more than two risk factors shows an OR of 3.83 [3.20–4.58]), and underlying conditions such as asthma (OR 6.70 [5.97–7.53]), nephropathies and hepatopathies (OR 6.64 [2.18–9.19]), diabetes (OR 3.95 [2.97–5.22]), hemoglobinopathies and clotting disorders (OR 3.28 [2.07–5.09]), immunosuppression and neoplasms (OR1.80 [0.68–4.48]), and miscellaneous diseases (OR 4.44 [2.18–9.19]). Obesity is negatively associated with influenza vaccination (OR 0.14 [0.13–0.16]).

In the multivariate analysis, the variables positively associated with vaccination were country of origin (non-native people were more positively associated with influenza vaccination) (OR 0.76 [0.63–0.91)], living in an urban area (OR 1.44 [1.25–1.64]), and having certain risk factors such as diabetes (OR 7.11 [2.03–4.5]), hemoglobinopathies and blood clotting disorders (OR 6.42 [1.72–23.4]), asthma (OR 6.12 [1.79–20.4]), neuromuscular and neurological diseases (OR 6.09 [1.43–25.0]), nephropathies and hepatopathies (OR 5.83 [1.38–23.7]), and miscellaneous diseases (OR 4.68 [1.29–16.8]). Both the immunosuppression and neoplasms variable and the number of risk factors lost statistical significance in this analysis.

## 4. Discussion

### 4.1. Discussion

Seasonal influenza represents a significant public health burden, and vaccination is the most effective strategy for preventing influenza infection [1,2,5,9]. Children are the main transmitters of the virus; therefore, more than 70 countries have implemented seasonal influenza vaccination programs for pediatric populations. However, differences exist between countries regarding the age groups included in routine vaccination schedules. For example, the United Kingdom has been vaccinating children aged 2 to 16 years against influenza since the 2013–2014 season, whereas Finland introduced influenza vaccination for children aged 6 to 36 months in 2007 and extended routine vaccination to children up to 6 years of age in 2015 [26,27,28].

In Spain, and consequently, in Catalonia, until the 2022–2023 season, influenza vaccination was recommended only for children aged 6 months and those older with underlying risk conditions [21,22]. From the 2023–2024 season onward, influenza vaccination was expanded to include all children aged 6 to 59 months [23,25]. In this way, influenza vaccination was considered routine in children of this age group. In older children (5–14 years) the vaccine continued to be administered only in those who had some underlying pathology [23]. This measure was applied following the recommendations of the Spanish Ministry of Health [23,25].

The data provided by our study indicate that, despite the application of this new measure, childhood influenza vaccination coverage (6 months to 14 years) remains very low, as follows: 18.1% and 19.3% for the 2023–24 and 2024–25 seasons, respectively. As for influenza vaccination in the range from 6 to 59 months, coverage was 19.1% during the 2023–24 season and 26.8% during the 2024–205 season.

Other local studies in Spain have shown that Galicia was the community with the most vaccinations, with a vaccine coverage of 55.8%, followed by Murcia (51.1%) and Andalusia (45.8%). Ceuta was the community with the lowest vaccination rates, with a coverage of 2.9%. Throughout Catalonia, influenza vaccination coverage was observed to be 24.4% [29,30,31]. It should be noted that these data, provided by the Ministry of Health, only include children aged 1 to 5 years (leaving out children aged 6 to 11 months), so our coverage is closer to the total figures recorded throughout the Catalan region. As for the 2024–25 season, official coverage figures at the state level have not yet been published for comparison, but a coverage of 58,6% has been registered this season in Andalusia for children from 6 to 59 months [32].

The reason for this heterogeneity between territories seems to be due to the different vaccination campaigns carried out in different autonomous communities [31]. For example, in Murcia and Andalusia, vaccinations took place in schools, while in Galicia, a mass vaccination campaign was promoted for a specific weekend in designated locations with longer vaccination shifts, imitating the COVID-19 vaccination campaign [32,33,34].

Another possible cause of the low coverage of influenza vaccination in children in our territory is the lack of promotional campaigns for influenza vaccination among health professionals. Several studies have shown that the main cause of influenza vaccination in children is advice from their pediatrician [9,35,36,37,38,39,40].

At the European level, influenza vaccination coverage varies considerably across countries. During the 2023–2024 season, the United Kingdom reported an influenza vaccination coverage of 50%. In Finland, coverage was 41% among children under 2 years of age and 29% among children aged 2 to 6 years. Denmark, which introduced universal influenza vaccination for children aged 2 to 6 years in the 2020–2021 season, reported a coverage rate of 22% [26,27,28,41].

However, it should be noted that, in the present study, we observed an increase in influenza vaccination during the 2024–25 season of 7.7% in the group from 6 to 59 months compared to the previous season, so we believe that once routine vaccination programs are more rooted in the territory, families will have more confidence in the initiative. This increase in coverage between seasons is also seen in Andalusia, where we see an increase between the two seasons of 12.8% [29].

It should be noted that this represents a substantial increase in coverage within the universally recommended group. In contrast, vaccination coverage among the risk-based target population (children with comorbidities) did not increase, underscoring the importance of universal vaccination strategies. Indeed, several studies have demonstrated that universal vaccination is the most cost-effective approach to reducing the overall burden of influenza at the population level [10,11,12,42,43,44,45,46].

Adherence to influenza vaccination during the last two flu seasons is very low. Only 17% of children have been vaccinated consecutively in the 2023–24 and 2024–25 seasons. At the Spanish level there is no study that analyzes these data in the last two seasons; therefore, we cannot assess whether our region is above or below the Spanish average. In a study carried out by our group in the five seasons prior to routine influenza vaccination programs in children under 5 years of age in Central Catalonia, we found an adherence to influenza vaccination of 21.6% in two seasons, a figure similar to our current results [14]. With these data we can say that the extension of influenza vaccination to children under 5 years of age has not led to an improvement in adherence. However, we would have to wait and see the trend in the coming seasons.

In relation to the variables associated with vaccination, our study shows that the male sex is more likely to be vaccinated than the female in the group from 6 to 59 months. In a study of Catalonia in 2015, Gonzalez R et al. also observed that gender was significantly associated with vaccination, but it appears not to be corroborated by most authors, so we do not consider it clinically relevant [13].

Regarding parent education, we have observed that both in the group of younger children (6 to 59 months) and in the group of 5- to 14-year-olds, parents with a higher educational level vaccinate more, probably because they have more information about influenza vaccination and the possible consequences of complications in case of an infection.

Non-native children are vaccinated more often than Spanish children. This result is similar to studies conducted in previous campaigns [13,14]. In a study conducted in Murcia during the 2022–23 campaign, Pérez Martin J. et al. found that there were no differences in influenza vaccination according to the children’s country of origin, but the parents’ country of origin did have a difference. This phenomenon can probably be explained by cultural reasons, but more studies are needed to reach a definitive conclusion.

The positive association between the number of risk factors and influenza vaccination, already described above [13,14], is quite logical. The higher the number of risk factors, the more likely it is to experience complications from influenza.

Finally, certain underlying conditions such as diabetes, asthma, heart disease, nephropathies and hepatopathies, and hemoglobinopathies and clotting disorders are also positively associated with influenza vaccination in children from 6 to 59 months.

Note that, in the 5 to 14-year-old age group, obesity is negatively associated with influenza vaccination; children with obesity are not vaccinated for the flu, probably because of their poor perception of illness. This is in line with a previous study conducted by our group [14].

### 4.2. Limitations and Strengths

The main limitation presented by our study is that the data are obtained from the ICS database; therefore, children vaccinated by other health providers were excluded from the study. However, the ICS is the main healthcare provider in Catalonia, and therefore, we believe that the data analyzed are representative of our population.

Another limitation of this study is the potential presence of incomplete data inherent to the use of medical and administrative databases. Specifically, information on parental educational attainment was missing for approximately half of the study population. This lack of completeness may have limited the ability to fully assess the association between parental education level and influenza vaccination uptake in children. However, given the large sample size and the population-based nature of the database, we believe that the overall findings remain robust and representative of the study population.

The main strength of our research is that this is one of the few studies that analyzes the factors associated with influenza vaccination in children under 5 years of age after the implementation of a routine influenza vaccination program in Spain. Another strength of the study is the sample size, as we present a sample of almost 40,000 children.

### 4.3. Futures Research

A possible future line of research could study the effect of the implementation of new campaigns to promote vaccination between the population and health professionals to try to increase the coverage of influenza vaccination in children, especially in the 6- to 59-month age group.

## 5. Conclusions

Influenza vaccination coverage and adherence among children in Central Catalonia remain low overall, despite the introduction of a routine influenza vaccination program for children aged 6–59 months from the 2023–2024 season onwards. However, a significant increase in vaccination coverage was observed in this universally recommended age group, rising from approximately 19% in the first season to nearly 27% in the second season. In contrast, vaccination coverage among children aged 5–14 years targeted through a risk-based recommendation remained low and showed no meaningful increase between seasons. These findings highlight the differential impact of universal versus risk-based influenza vaccination strategies, suggesting that universal recommendations may be more effective in improving coverage in pediatric populations. Factors associated with influenza vaccination in children aged 6–59 months included immigrant origin, urban residence, the presence of multiple risk factors, and specific underlying chronic conditions. Continued efforts are needed to strengthen influenza vaccination programs, particularly among children targeted through risk-based strategies, to improve both coverage and adherence.

## Figures and Tables

**Table 1:** High-risk pathology that the Catalan Department of Health considered as indications for receiving the influenza vaccine in children older than 6 months up to the 2022/23 season [14].

High-Risk Pathology for Influenza Vaccination in Children
- Chronic cardiovascular, neurological, or respiratory diseases (including hypertension, asthma, bronchopulmonary dysplasia, and cystic fibrosis). - Diabetes mellitus. - Morbid obesity: body mass index (BMI) ≥ 35 in adolescents and ≥3 standard deviations in children. - Chronic kidney disease and nephrotic syndrome. - Hemoglobinopathies and anemia. - Hemophilia and other clotting disorders, chronic bleeding disorders, recipients of blood products, and multiple transfusions. - Asplenia or severe splenic dysfunction. - Chronic liver disease. - Severe neuromuscular diseases. - Immunosuppression (including primary immunodeficiencies and those caused by HIV infection), drugs (including eculizumab treatment), or in transplant recipients and associated deficiencies. - Cancer and malignant blood diseases. - Cochlear implant or awaiting implant. - Cerebrospinal fluid fistula. - Coeliac disease. - Chronic inflammatory disease. - Disorders and diseases that entail cognitive dysfunction: Down’s syndrome, etc. - Children and adolescents receiving prolonged treatment with acetylsalicylic acid, due to the possibility of developing Reye’s syndrome after influenza. - Long-term institutionalized children. - Children between 6 months and 2 years with a history of prematurity, born at less than 32 weeks’ gestation.

**Table 2:** Clinical and demographic characteristics of children eligible for the influenza vaccination during the 2023–24 and 2024–25 seasons.

Total (N = 39,987)	Variables
	**Age:**
16,003 (40.0%)	6 months–2 years
14,123 (35.3%)	3–5 years
4430 (11.1%)	6–10 years
5431 (13.6%)	11–14 years
	**Father’s level of education:**
240 (0.6%)	No education
3918 (9.8%)	Primary
4115 (10.3%)	Secondary (lower)
3475 (8.7%)	Higher secondary
5213 (13.0%)	University/Third level
306 (0.8%)	Other
22,720 (57%)	No answer
	**Mother’s level of education:**
367 (0.9%)	No education
2732 (6.8%)	Primary
3490 (8.7%)	Secondary(lower)
3481 (8.7%)	Higher secondary
7513 (18.8%)	University/Third level
193 (0.5%)	Other
22,211 (55.5%)	No answer
	**Flu vaccination:**
31,621 (79.1%)	No
8366 (20.9%)	Yes
	**Season:**
20,348 (50.9%)	2023–2024
19,639 (49.1%)	2024–2025
	**Number of risk factors:**
28,954 (72.4%)	0
10,461 (26.2%)	1
557 (1.4%)	2
15 (0.04%)	3
	**Risk factors (1, 2 and 3):**
3220 (8%)	Asthma
591 (1.5%)	Heart disease
43 (0.1%)	Chromosomopathies and metabolopathies
225 (0.6%)	Diabetes
9 (0.02%)	Hemoglobinopathies and clotting disorders
155 (0.34%)	Immunosuppression and neoplasms
42 (0.1%)	Neuromuscular and neurological diseases
726 (1.8%)	Celiac disease
167 (0.4%)	Miscellaneous
57 (0.1)	Nephropathies and hepatopathies
6272 (15.6%)	Obesity

**Table 3:** Descriptive and comparative analysis between vaccinated and unvaccinated children between 6 and 59 months.

Children from 6 to 59 Months			
***p*** **-Value**	Vaccinated (N = 6794)	Not Vaccinated (N = 22,936)	
<0.001			**Age:**
	4395 (64.6%)	11,608 (50.6%)	6 months–24 months
	2399 (35.3%)	11,328 (49.4%)	25 months–59 months
	-	-	5–10 years
	-	-	11–14 years
<0.001			**Father’s level of education:**
0.968	37 (0.5%)	124 (0.5%)	No education
<0.001	718 (10.6%)	1846 (8.05%)	Primary
<0.001	833 (12.3%)	2476 (10.8%)	Secondary (lower)
<0.033	681 (10.0%)	2103 (9.2%)	Higher secondary
<0.001	1116 (16.4%)	3241 (14.1%)	University/Third level
0.058	62 (0.9%)	158 (0.7%)	Other
<0.001	3347 (49.3%)	12,988 (56.6%)	No answer
<0.001			**Mother’s level of education:**
0.092	68 (1.00%)	181 (0.8%)	No education
<0.001	566 (8.3%)	1280 (5.6%)	Primary
<0.001	654 (9.6%)	1930 (8.4%)	Secondary (lower)
0.004	682 (10.0%)	2045 (8.9%)	Higher secondary
<0.001	1544 (22.7%)	4726 (20.6%)	University/High School
0.978	33 (0.5%)	112 (0.5%)	Other
<0.001	3247 (47.8%)	12,662 (55.2%)	No answer
<0.001			**Number of risk factors:**
<0.001	6512 (95.8%)	22,441 (97.8%)	0
<0.001	274 (4.03%)	488 (2.1%)	1
0.004	8 (0.1%)	7 (0.03%)	≥2
			**Risk factors:**
<0.001	71 (1.0%)	67 (0.3%)	Asthma
0.002	68 (1.00%)	145 (0.6%)	Heart disease
1	2 (0.02%)	9 (0.03%)	Chromosomopathies and metabolopathies
0.003	9 (0.1%)	7 (0.03%)	Diabetes
<0.001	13 (0.2%)	10 (0.04%)	Hemoglobinopathies and clotting disorders
0.112	11 (0.0%)	19 (1%)	Immunosuppression and neoplasms
<0.001	5 (1%)	0 (0.00%)	Neuromuscular and neurological diseases
0.074	20 (0.3%)	40 (0.2%)	Celiac disease
<0.001	24 (0.4%)	20 (0.1%)	Miscellaneous
0.004	7 (0.1%)	4 (0.01%)	Nephropathies and hepatopathies
0.495	60 (0.9%)	181 (0.8%)	Obesity
<0.001			**Season:**
	2916 (42.9%)	12,356 (53.9%)	2023–2024
	3878 (57.1%)	10,580 (46.1%)	2024–2025

**Table 4:** Descriptive and comparative analysis between vaccinated and unvaccinated children between 5 and 14 years.

Children from 5 to 14 Years			
*p*-Value	Vaccinated (1572)	Not Vaccinated (N = 8685)	
<0.001			**Age:**
	-	-	6 months–24 months
	-	-	25 months–59 months
	814 (51.8%)	4012 (46.2%)	5–10 years
	758 (48.2%)	4673 (53.8%)	11–14 years
0.012			**Father’s level of education:**
0.003	7 (0.45%)	72 (0.8%)	No education
<0.001	168 (10.7%)	1186 (13.7%)	Primary
<0.001	114 (7.2%)	692 (8%)	Secondary (lower)
<0.001	110 (7.0%)	581 (6.7%)	Higher secondary
0.003	7 (0.5%)	72 (0.8%)	University/Third level
<0.001	16 (1.0%)	70 (0.8%)	Other
<0.001	1012 (64.4%)	5373 (61.9%)	No answer
0.008			**Mother’s level of education:**
0.001	12 (0.8%)	106 (1.2%)	No education
<0.001	132 (8.4%)	754 (8.7%)	Primary
<0.001	107 (6.8%)	799 (9.2%)	Secondary (lower)
<0.001	109 (6.9%)	645 (7.4%)	Higher secondary
<0.001	212 (13.5%)	1031 (11.9%))	University/High School
0.016	4 (0.2%)	44 (0.5%)	Other
<0.001	996 (63.4%)	5306 (61.1%)	No answer
<0.001			**Number of risk factors:**
0.586	0 (0.0%)	1 (0.01%)	0
<0.001	1358 (86.4%)	8341 (96.0%)	1
<0.001	214 (13.6%)	343 (3.95%)	≥2
			**Risk factors:**
<0.001	1055 (67.1%)	2027 (23.3%)	Asthma
0.949	57 (3.6%)	321 (3.7%)	Heart disease
0.217	2 (0.1%)	30 (0.3%)	Chromosomopathies and metabolopathies
<0.001	85 (5.4%)	124 (1.4%)	Diabetes
<0.001	31 (2%)	53 (0.6%)	Hemoglobinopathies and clotting disorders
0.012	30 (1.9%)	97 (1.1%)	Immunosuppression and neoplasms
<0.001	15 (1%)	22 (0.2%)	Neuromuscular and neurological diseases
0.032	122 (7.8%)	545 (6.3%)	Celiac disease
<0.001	35 (2.2%)	88 (1.0%)	Miscellaneous
<0.001	17 (2%)	29 (0.3%)	Nephropathies and hepatopathies
<0.001	345 (21.9%)	5698 (65.6%)	Obesity
0.495			**Season:**
	765 (48.7%)	4311 (49.6%)	2023–2024
	807 (51.3%)	4374 (50.4%)	2024–2025

**Table 5:** General influenza vaccination coverage by season throughout pediatric age (children from 6 months to 14 years).

IC 95%	Diff %	Coverage in %	Vaccinated/Eligible for Vaccination	Season
		18.1	3.681/20.348	2023–2024
[−0.014; −0.002]	1.2	19.3	4.685/24.324	2024–2025

**Table 6:** Influenza vaccination coverage in the 6- to 59-month age range.

IC 95%	Diff %	Coverage in %	Vaccinated/Eligible for Vaccination	Season
		19.1	2916/15,272	2023–2024
[−0.059; −0.043]	7.7	26.8	3878/14,458	2024–2025

**Table 7:** Influenza vaccination coverage in the 5- to 14-year-old age range (children with risk pathologies).

IC 95%	Diff %	Coverage in %	Vaccinated/Eligible	Season
		17.8	765/4310	2023–2024
[−0.018; 0.008]	0.7	18.5	807/4374	2024–2025

**Table 8:** Bivariate and multivariate analysis of the association of the different variables with influenza vaccination in the 6–59 month group. Reference: Not having the risk factor.

Children from 6 to 59 Months				
Multivariate Analysis		Bivariate Analysis		
*p*-Value	OR (IC 95%)	*p*-Value	OR (IC 95%)	
				**Number of risk factors:**
Ref.	Ref.	Ref.	Ref.	0
<0.001	2.74 [1.91; 3.85]	<0.001	1.93 [1.66; 2.25]	1
0.062	5.82 [0.83; 37.3]	0.008	3.94 [1.41; 11.2]	≥2
				**Risk factors:**
<0.001	2.38 [1.40; 4.07]	<0.001	3.60 [2.58; 5.05]	Asthma
0.01	0.43 [0.22; 0.81]	0.001	1.59 [1.18; 2.11]	Heart disease
0.943	0.00 [-; 4.86]	0.713	0.75 [0.11; 2.91]	Chromosomopathies and metabolopathies
0.01	4.43 [1.40; 14.5]	0.003	4.34 [1.62; 12.2]	Diabetes
0.107	2.34 [0.79; 6.59]	<0.001	4.40 [1.93; 10.3]	Hemoglobinopathies and clotting disorders
0.699	1.24 [0.36; 3.64]	0.076	1.96 [0.90; 4.05]	Immunosuppression and neoplasms
-	-	-	-	Neuromuscular and neurological diseases
0.901	0.94 [0.39; 2.10]	0.055	1.69 [0.96; 2.86]	Celiac disease
0.071	2.20 [0.90; 5.15]	<0.001	4.06 [2.24; 7.43]	Miscellaneous
0.019	5.83 [1.36; 29.6]	0.004	5.91 [1.79; 22.6]	Nephropathies and hepatopathies
-	-	0.448	1.12 [0.82; 1.49]	Obesity
				**Mother’s level of education:**
0.075	0.44 [0.17; 1.05]	0.265	0.63 [0.26; 1.37]	Other
0.839	0.94 [0.57; 1.63]	0.535	0.86 [0.56; 1.40]	Higher secondary
0.65	1.12 [0.68; 1.93]	0.366	1.24 [0.79; 2.00]	Primary
0.804	0.93 [0.56; 1.61]	0.455	0.84 [0.54; 1.36]	Secondary (lower)
Ref.	Ref.	Ref.	Ref.	No education
0.742	0.91 [0.52; 1.63]	0.271	0.78 [0.51; 1.24]	No answer
0.626	0.87 [0.53; 1.51]	0.309	0.79 [0.52; 1.27]	University/High School.
				**Father’s level of education:**
0.006	3.17 [1.43; 7.64]	0.033	2.35 [1.11; 5.47]	Other
0.39	1.38 [0.69; 3.10]	0.436	1.32 [0.69; 2.83]	Higher secondary education
0.1	1.83 [0.93; 4.06]	0.078	1.86 [0.98; 3.98]	Primary
0.401	1.37 [0.68; 3.05]	0.354	1.39 [0.73; 2.97]	Secondary (lower)
Ref.	Ref.	Ref.	Ref.	No education
0.273	1.53 [0.74; 3.53]	0.362	1.37 [0.73; 2.91]	No answer
0.137	1.75 [0.87; 3.91]	0.192	1.58 [0.84; 3.37]	University/High School

**Table 9:** Bivariate and multivariate analysis of the association of the different variables with influenza vaccination in the 5- to 14-year-old group. Reference: Not having the risk factor.

Children Between 5 and 14 Years				
Multivariate Analysis		Bivariate Analysis		
*p*-Value	OR (IC 95%)	*p*-Value	OR (IC 95%)	
				**Number of risk factors:**
-	-	-	-	0
Ref.	Ref.	Ref.	Ref.	1
0.247	2.09 [0.59; 7.50]	<0.001	3.83 [3.20; 4.58]	≥2
				**Risk factors:**
0.002	6.25 [1.83; 20.9]	<0.001	6.70 [5.97; 7.53]	Asthma
0.454	1.58 [0.45; 5.28]	0.892	0.98 [0.72; 1.30]	Heart disease
0.6	0.58 [0.06; 3.86]	0.17	0.36 [0.05; 1.22]	Chromosomopathies and metabolopathies
0.001	7.47 [2.13; 25.7]	<0.001	3.95 [2.97; 5.22]	Diabetes
0.004	6.42 [1.72; 23.4]	<0.001	3.28 [2.07; 5.09]	Hemoglobinopathies and clotting disorders
0.148	2.51 [0.69; 8.78]	0.009	1.72 [1.12; 2.57]	Immunosuppression and neoplasms
0.013	5.98 [1.41; 24.5]	<0.001	3.79 [1.93; 7.27]	Neuromuscular and neurological diseases
0.183	2.27 [0.65; 7.65]	0.028	1.26 [1.02; 1.54]	Celiac disease
0.012	5.02 [1.36; 18.1]	<0.001	2.22 [1.48; 3.27]	Miscellaneous
0.023	5.06 [1.20; 20.6]	<0.001	3.26 [1.75; 5.89]	Nephropathies and hepatopathies
0.174	0.43 [0.12; 1.45]	<0.001	0.14 [0.13; 0.16]	Obesity
				**Mother’s level of education:**
Ref.	Ref.	0.717	0.80 [0.21; 2.45]	Other
0.049	5.06 [1.22; 35.5]	0.213	1.49 [0.82; 2.94]	Higher secondary
0.025	6.31 [1.53; 44.3]	0.172	1.55 [0.86; 3.03]	Primary
0.086	4.15 [0.99; 29.3]	0.601	1.18 [0.65; 2.33]	Secondary (lower)
0.195	3.14 [0.64; 23.8]	Ref.	Ref.	No education
0.088	4.18 [0.97; 30.0]	0.099	1.66 [0.94; 3.19]	No answer
0.045	5.15 [1.25; 36.0]	0.057	1.82 [1.02; 3.53]	University/High School.
				**Father’s level of education:**
Ref.	Ref.	0.076	2.35 [0.94; 6.44]	Other
0.064	0.46 [0.20; 1.07]	0.103	1.95 [0.93; 4.75]	Higher secondary education
0.011	0.35 [0.16; 0.81]	0.352	1.46 [0.70; 3.53]	Primary
0.017	0.37 [0.16; 0.86]	0.197	1.69 [0.81; 4.13]	Secondary (lower)
0.106	0.37 [0.11; 1.21]	Ref.	Ref.	No education
0.155	0.52 [0.22; 1.30]	0.096	1.94 [0.95; 4.64]	No answer
0.008	0.33 [0.15; 0.77]	0.068	2.10 [1.01; 5.10]	University/High School

## Data Availability

The original contributions presented in this study are included in the article. Further inquiries can be directed to the corresponding author.
